# Replacement of l-Amino Acids by d-Amino Acids in the Antimicrobial Peptide Ranalexin and Its Consequences for Antimicrobial Activity and Biodistribution

**DOI:** 10.3390/molecules24162987

**Published:** 2019-08-17

**Authors:** Cornelius Domhan, Philipp Uhl, Christian Kleist, Stefan Zimmermann, Florian Umstätter, Karin Leotta, Walter Mier, Michael Wink

**Affiliations:** 1 Institute of Pharmacy and Molecular Biotechnology, Heidelberg University, 69120 Heidelberg, Germany; 2 Department of Nuclear Medicine, Heidelberg University Hospital, 69120 Heidelberg, Germany; 3 Department of Infectious Diseases, Medical Microbiology and Hygiene, Heidelberg University Hospital, 69120 Heidelberg, Germany

**Keywords:** Ranalexin, peptide therapeutics, antibiotics, configuration, antimicrobial activity

## Abstract

Infections caused by multidrug-resistant bacteria are a global emerging problem. New antibiotics that rely on innovative modes of action are urgently needed. Ranalexin is a potent antimicrobial peptide (AMP) produced in the skin of the American bullfrog *Rana catesbeiana*. Despite strong antimicrobial activity against Gram-positive bacteria, ranalexin shows disadvantages such as poor pharmacokinetics. To tackle these problems, a ranalexin derivative consisting exclusively of d-amino acids (named danalexin) was synthesized and compared to the original ranalexin for its antimicrobial potential and its biodistribution properties in a rat model. Danalexin showed improved biodistribution with an extended retention in the organisms of Wistar rats when compared to ranalexin. While ranalexin is rapidly cleared from the body, danalexin is retained primarily in the kidneys. Remarkably, both peptides showed strong antimicrobial activity against Gram-positive bacteria and Gram-negative bacteria of the genus *Acinetobacter* with minimum inhibitory concentrations (MICs) between 4 and 16 mg/L (1.9–7.6 µM). Moreover, both peptides showed lower antimicrobial activities with MICs ≥32 mg/L (≥15.2 µM) against further Gram-negative bacteria. The preservation of antimicrobial activity proves that the configuration of the amino acids does not affect the anticipated mechanism of action, namely pore formation.

## 1. Introduction

Bacteria that possess multidrug-resistance against common antibiotics are spreading worldwide [1]. Infections with bacteria cause increasing numbers of deaths and thus endanger the achievements of modern medicine [2]. To circumvent a return to a pre-antibiotic state, innovative antibiotics are urgently needed. 

Antimicrobial peptides (AMPs) are highly effective, amphiphilic, cationic peptides produced by a wide variety of lifeforms [3]. Ranalexin is an AMP that is produced in the skin of the North American bullfrog *Rana catesbeiana* [4]. This peptide of 20-amino acid length has strong antimicrobial activity against Gram-positive bacteria, and its efficacy was previously shown in animal infection models [5]. So far, no host-defense peptide has been approved as an antibiotic drug [6]. Because of their amino acid backbone, AMPs possess intrinsic weaknesses such as a short plasma half-life and degradability by proteolytic enzymes [7,8]. 

Peptide bonds formed by d-amino acids are resistant to degradation by proteolytic enzymes [9]. A specific substitution of l-amino acids would impair the antimicrobial activity of an AMP [10]. Therefore, we substituted all l-amino acids of ranalexin with d-amino acids (hereinafter named danalexin). Danalexin and ranalexin were tested for their antimicrobial activity against a broad variety of bacteria, including multidrug-resistant pathogens. Further, the antimicrobial kinetics of both substances were investigated by time-kill curves. The biodistribution of danalexin in a rat model was investigated by scintigraphy and positron emission tomography (PET). For both in vivo imaging modalities, specially designed tracer peptides were required. For scintigraphy, an additional d-tyrosine was coupled to danalexin (d-Tyr-danalexin), whereas for PET imaging, the chelating moiety DOTA was covalently attached to danalexin (DOTA-d-Tyr-danalexin). Amino acid sequences of the synthesized peptides are shown in Table 1. 

We found that danalexin retains the spectrum of antibacterial efficacy of ranalexin. The antimicrobial time-kill kinetics of both substances were comparable. Furthermore, in vivo imaging of danalexin was found to be superior to that of ranalexin, because of accumulation and prolonged retention in the kidneys.

## 2. Results

### 2.1. Peptide Synthesis

Pure batches of the peptides were obtained by peptide synthesis. Their calculated molecular masses and the results of the HPLC-MS analyses are shown in Table 2, proving that the synthesis was correct.

### 2.2. Antimicrobial Susceptibility of Clinical Isolates

The results of the antimicrobial susceptibility testing of clinical isolates from Heidelberg University Hospital are shown in Table 3. The results of the antimicrobial testing of the clinical isolate *Acinetobacter baumannii* SC322333 has been published before [11]. In *A. baumannii* SC411190, a *bla*_OXA-23_ resistance gene was found, coding for a carbapenemase enzyme. *Enterococcus faecium* UL407074 contains a *vanA* resistance gene. *Klebsiella pneumoniae* BL809453 contains a *bla*_KPC_ resistance gene, coding for a KPC-2 (*K. pneumoniae* carbapenemase) enzyme. All three Gram-negative clinical isolates belong to the 4-MRGN (multidrug-resistant Gram-negative bacteria) group. The results substantiate that colistin often constitutes the last-line antibiotic against multidrug-resistant Gram-negative infections.

### 2.3. Antimicrobial Activity of Ranalexin and Danalexin

The results of antimicrobial testing are documented in Table 4. All experiments were performed in triplicates in three independent experiments. Ranalexin and danalexin showed a similar spectrum of antimicrobial activity. Both peptides have strong antimicrobial activity against Gram-positive bacteria and Gram-negative bacteria of the genus *Acinetobacter* with minimum inhibitory concentrations (MICs) in the range of 4–16 mg/L. Against other Gram-negative bacteria, only weak antimicrobial activity (32–>64 mg/L) could be observed. The activities of ranalexin and danalexin were comparable.

### 2.4. Time-Kill Curves

**Figure 1:**
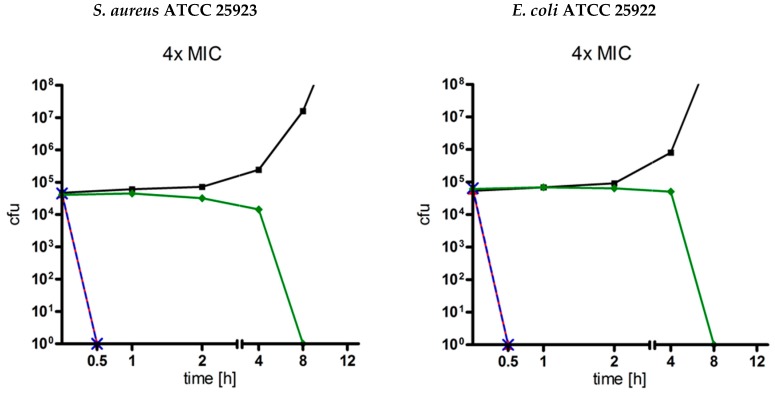

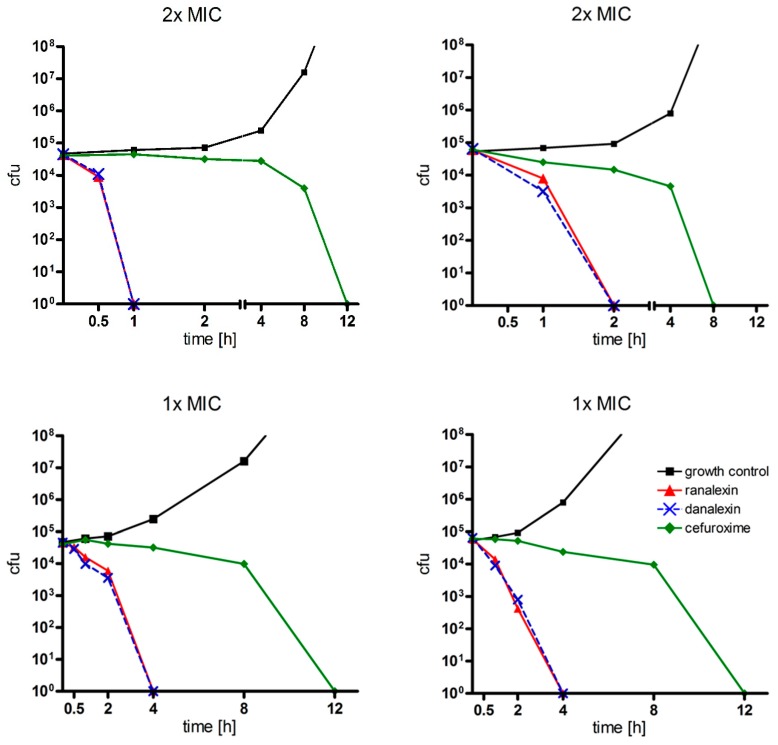
Time-kill curves of ranalexin and danalexin (*n* = 1). Time-kill curves were determined at 1×, 2× and 4× MIC against *E. coli* ATCC 25922 and *S. aureus* ATCC 25923. Ranalexin (red) and danalexin (blue) showed a fast, concentration-dependent mode of action. Both substances were more bactericidal compared to cefuroxime (green).

For the estimation of antimicrobial kinetics, time-kill curves of ranalexin and danalexin against the well examined Gram-positive bacterium *S. aureus* ATCC 25923 and the Gram-negative bacterium *E. coli* ATCC 25922 were performed (*n* = 1). Results are displayed in Figure 1. Ranalexin and danalexin showed fast, concentration-dependent time-kill kinetics. Between danalexin und ranalexin, no difference in antibacterial kinetics could be observed. At concentrations of 4× MIC, no living bacteria could be detected after 30 min of incubation. At concentrations of 1× MIC, no living bacteria could be detected after 4 h of incubation. Both substances were superior when compared to the established cephalosporin antibiotic cefuroxime, which needed concentration-dependent 8–12 h until no living bacteria could be detected. Cefuroxime was selected as a control compound because it is active against both strains tested.

### 2.5. Scintigraphy

**Figure 2:**
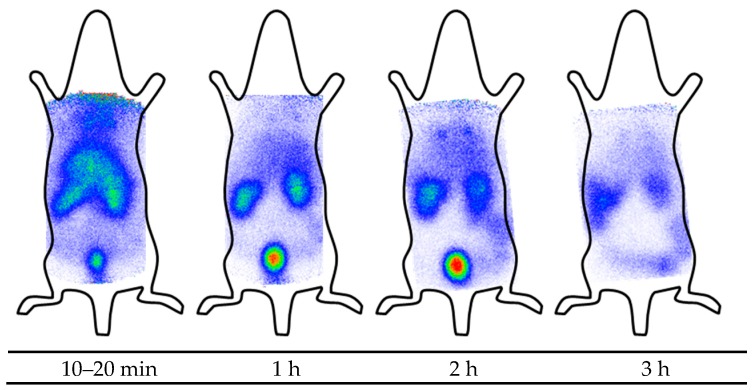
Scintigraphic imaging of ranalexin-d-Tyr in rats. Images were recorded 10–20 min, 1 h, 2 h and 3 h post injection into the tail vein of a Wistar rat. The peptide is excreted by the kidneys. At 1 h post injection, the majority of the substance is found in the bladder.

**Figure 3:**
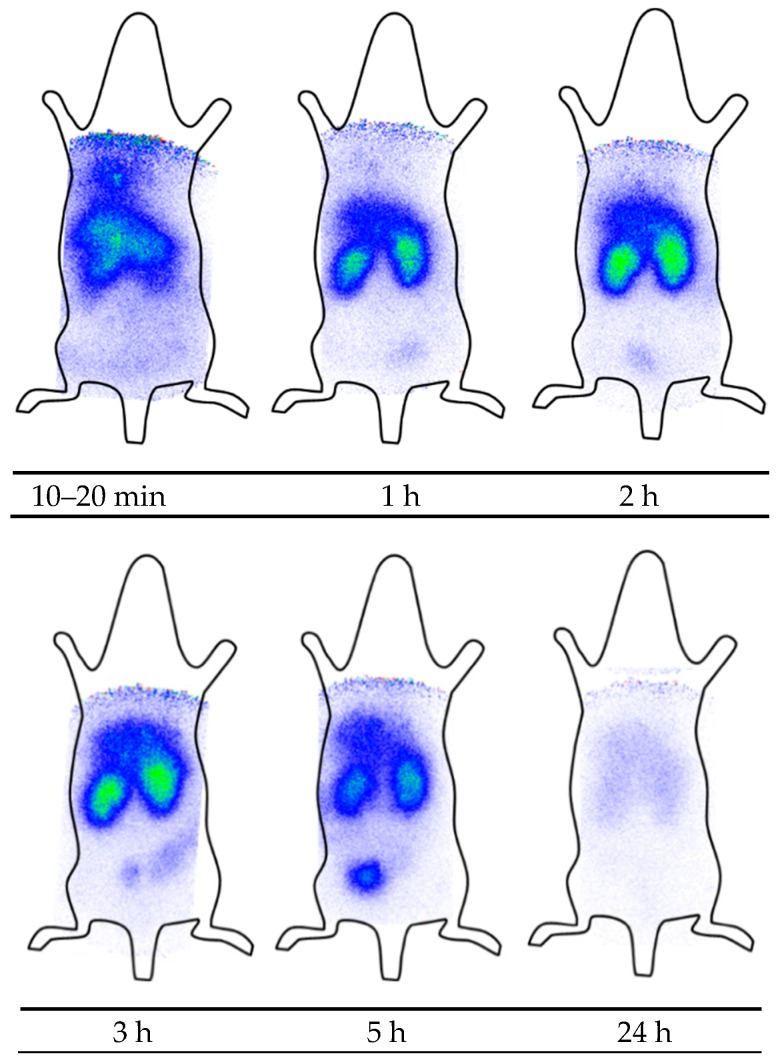
Scintigraphic images of d-Tyr-danalexin. Images were recorded 10–20 min, 1 h, 2 h, 3 h, 5 h and 24 h post injection into the tail vein of a Wistar rat. An accumulation of d-Tyr-danalexin in the kidneys is clearly visible. Even 24 h post injection, radioactivity is still visible in the kidneys.

To gain insight into the in vivo behavior of the AMPs, scintigraphic images of ^125^I-labeled d-Tyr-danalexin and ranalexin-d-Tyr were obtained after intravenous injection into the tail vein of a Wistar rat. Scintigraphy images of ranalexin-d-Tyr are shown in Figure 2. The images clearly indicate that ranalexin-d-Tyr is predominantly detected in the kidneys. Additionally, smaller amounts of ranalexin-d-Tyr are found in the liver. The excretion occurs rapidly via the urine. Three hours post injection, the vast majority of the substance is already excreted. Minor amounts can be found in the kidneys and in the gut.

d-Tyr-danalexin shows a superior biodistribution because of prolonged renal retention when compared to ranalexin, so it might be applied for the treatment of renal infections (Figure 3). The peptide is mainly distributed in the kidneys and accumulates there for a minimum of 5 h. Even 24 h post injection, d-Tyr-danalexin can be found in the kidneys. Small amounts are distributed in the liver. The excretion takes place via the urine.

### 2.6. Micro-PET Imaging

**Figure 4:**
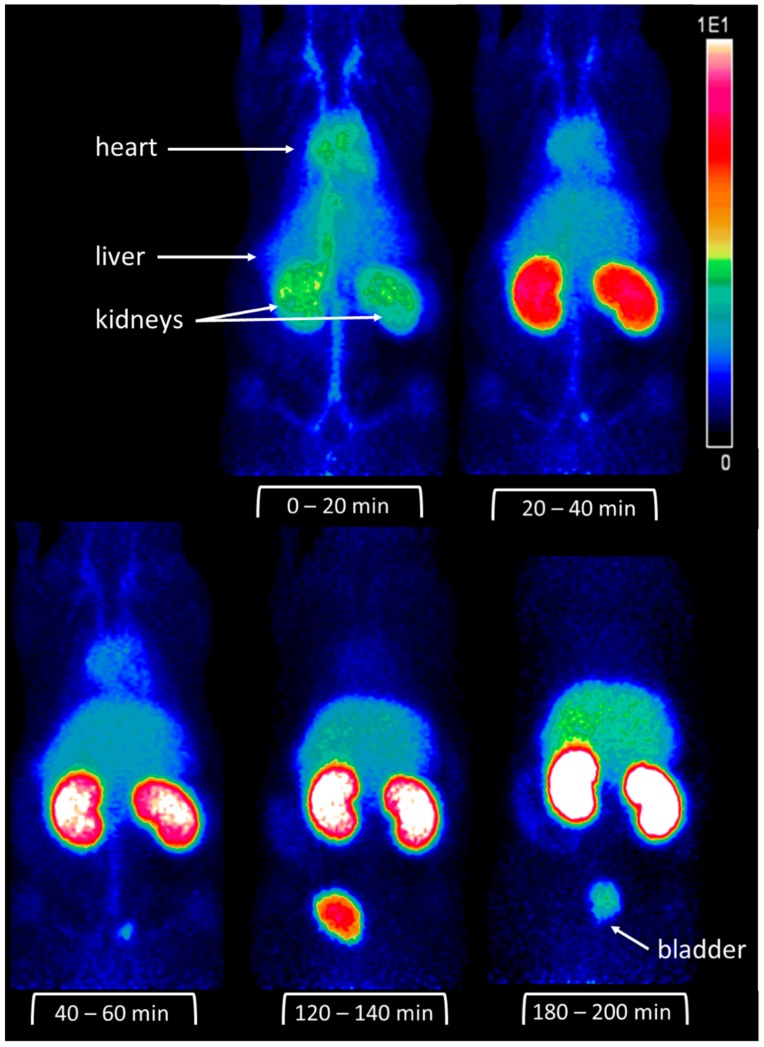
Positron emission tomography (PET) images of DOTA-d-Tyr-danalexin in a Wistar rat. After injection into the tail vein, distribution in the circulation (as reflected by the perfusion of the heart, kidneys, liver and the blood vessels) is visible. At 20 min after injection, an accumulation in the kidneys is clearly visible. This accumulation in the kidneys remains for at least 3 h. Smaller amounts of DOTA-d-Tyr-danalexin are taken up by the liver.

**Figure 5:**
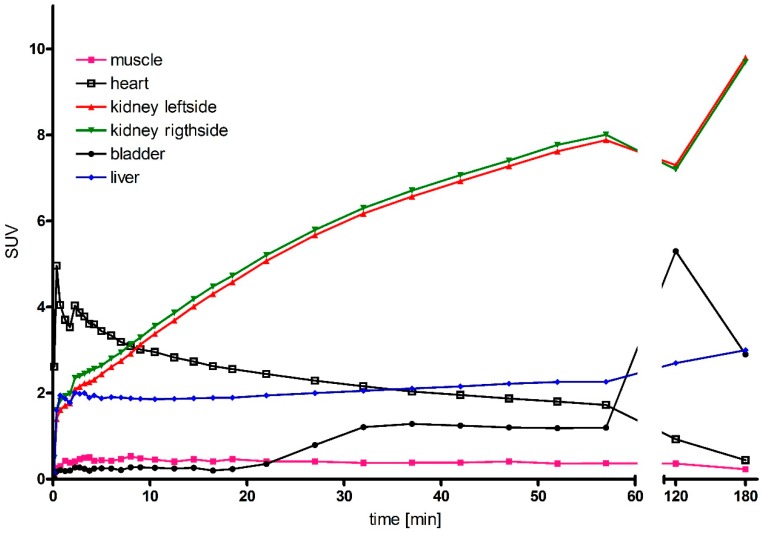
Standard uptake values (SUVs) of DOTA-d-Tyr-danalexin after injection into the tail vein of a Wistar rat. The SUV is used for the quantification of radioactivity in the individual organs. The accumulation of the substance in the kidneys is clearly visible. Smaller amounts are found in the liver. DOTA-d-Tyr-danalexin is excreted via the bladder.

For higher resolution images, PET imaging of ^68^Ga-labelled DOTA-d-Tyr-danalexin was performed. The images are shown in Figure 4. For comparison, the PET images of DOTA-ranalexin were published previously [11]. After injection into the tail vein of a Wistar rat, DOTA-d-Tyr-danalexin is distributed in the heart, liver and kidneys. At 20 min post injection, accumulation in the kidneys dominates. Even 3 h post injection, enhanced accumulation in the kidneys is visible. Excretion takes place via the urine. Standard uptake values (SUVs) are shown in Figure 5, substantiating the accumulation of DOTA-d-Tyr-danalexin in the kidneys.

## 3. Discussion

In this study, the influence of the substitution of proteinogenic amino acids on the antimicrobial activity and biodistribution of the AMP ranalexin was investigated. Solid phase peptide synthesis provided a suitable method for manufacturing peptides containing d-amino acids [13]. HPLC-MS analysis confirmed the purity of the obtained peptides. Ranalexin is an intensively investigated AMP, known for strong antimicrobial activity against Gram-positive bacteria with a rapid bactericidal mode of action [4,14]. The short plasma half-life in vivo is a general disadvantage of AMPs consisting of L-amino acids [8]. Truncated lipopeptide derivatives of ranalexin were found to overcome problems such as short plasma half-life and lack of activity against Gram-negative bacteria [11]. An all d-amino acid derivative was synthesized, because the substitution of only a few amino acids would change the secondary structure of the peptide, eventually leading to a loss of function [10,15]. In cases of AMPs with unspecific antibacterial modes of action, the substitution of all l-amino acids by d-amino acids might allow the retention of their antimicrobial activity [16]. A ranalexin derivative consisting exclusively of d-amino acids had previously been synthesized. Unfortunately, in this peptide, the L-isoleucine moieties had been substituted by d-leucine due to cost reasons [17]. In the peptide designed for this study, the original isoleucine was maintained—and thus incorporated as d-isoleucine—to prevent potential influences of a changed amino acid on the AMP conformation. Danalexin was found to retain the antimicrobial spectrum of ranalexin, including its activity against Gram-positive bacteria and, remarkably, Gram-negative bacteria of the genus *Acinetobacter*. This could be explained by the composition of the outer membranes of these bacteria, which contain higher amounts of carbohydrates and lower amounts of lipopolysaccharides when compared to other Gram-negative bacteria [18]. There were no or negligible differences in MICs between ranalexin and danalexin on the strains tested. This finding sustains the assumption of an unspecific membrane-targeted mode of action [16]. A specific mode of action would most probably be influenced by the conformation of the amino acids. For ranalexin, a rapid, bactericidal mode of action had already been shown [14]. The time-kill curves of ranalexin and danalexin revealed that both substances show similar fast antibacterial kinetics, which also underlines their unspecific mode of action. Compared to previously performed time-kill curves of ranalexin, herein minor differences occurred, because only one set of experiments was carried out due to the laborious and costly character of performing time-kill curves. Notably, both peptides showed superior antimicrobial kinetics when compared to the established bactericidal cefuroxime. Another important objective of this study was the investigation of the effect of the d-amino acids on the in vivo behavior of the modified AMP. Naturally occurring AMPs consisting of l-amino acids suffer from the disadvantage of a short plasma half-life and rapid renal clearance [8]. For ranalexin, a rapid renal clearance was shown before [11]. For non-proteinogenic amino acid-containing peptides such as polymyxins, an accumulation in the kidneys has previously been reported due to their stability against proteolytic degradation [19]. Both scintigraphy and micro-PET imaging revealed that the exchange with d-amino acids leads to a prolonged retention of the peptide danalexin (or its degradation products) in the kidneys. 

## 4. Material and Methods

### 4.1. Peptide Synthesis

Peptides were manufactured by solid-phase peptide synthesis using the Fmoc strategy on an Applied Biosystems 433A synthesizer (Thermo Fisher Scientific, Darmstadt, Germany) [20]. For coupling of the chelator 1,4,7,10-tetraazacyclododecane-1,4,7,10-tetraacetic acid (DOTA), Fmoc-Lys(alloc) as C-terminal lysine was used [21]. Cleavage from the resin was achieved by trifluoroacetic acid (TFA, Biosolve, Valkenswaard, The Netherlands), as described previously by Brings et al. [21]. Disulfide bridges were formed by dropwise addition of 30 mg/mL iodine (Merck, Darmstadt, Germany) in acetic acid (Sigma-Aldrich, Steinheim, Germany). Excessive iodine was inactivated with ascorbic acid (Merck, Darmstadt, Germany). Purification of the peptides was achieved by preparative HPLC on a Gilson 321 high-performance liquid chromatography (HPLC) system with a Reprosil Gold 120 C18 4-μm 150 × 120 mm column (Dr. Maisch HPLC, Ammerbuch, Germany) [20]. The purity of the peptides was determined by HPLC-MS using an Exactive Orbitrap system (Thermo Fisher Scientific, Bremen, Germany) equipped with a C18 column (Hypersil Gold aQ, Thermo Fisher).

### 4.2. Antimicrobial Activity

*Bacillus megaterium* DSM 32, *Bacillus subtilis* DSM 10, *Clostridium pasterianum* DSM 525, *Corynebacterium spheniscorum* DSM 44757, *Pseudomonas fluorescens* DSM 50090 and Yersinia mollaretii DSM 18520 were purchased from the Leibniz Institute DSMZ (Deutsche Sammlung von Mikroorganismen und Zellkulturen - German Collection of Microorganisms and Cell Cultures, Braunschweig, Germany). Other bacteria were obtained from the Department of Medical Microbiology and Hygiene, Heidelberg University Hospital (Heidelberg, Germany). Minimum inhibitory concentrations (MICs) were determined by broth microdilution according to European Committee on Antimicrobial Susceptibility Testing (EUCAST) [22]. In detail, the protocol was described previously [11]. For *C. spheniscorum*, cation-adjusted Mueller–Hinton broth supplemented with 5% lysed horse blood was used. For *C. pasterianum*, supplemented Brucella Broth according to Clinical and Laboratory Standards Institute (CLSI) was used [23]. The anaerobic atmosphere for the incubation of *C. pasterianum* was generated with Anaerocult® C mini systems (Merck, Darmstadt, Germany). After 20 h of incubation at 35 +/− 1 °C, respectively, 30 +/− 1 °C for *B. subtilis, B. megaterium, P. fluorescens* and *Y. mollaretii*, MIC was determined as the lowest concentration without visible growth. For *C. spheniscorum* and *C. pasterianum*, 40 h of incubation was necessary to obtain significant growth. As positive controls, vancomycin (potency 994 µg/mg; Sigma-Aldrich, Steinheim, Germany), respectively, colistin (potency 753 µg/mg; Carl Roth, Karlsruhe) or doxycycline (potency 842 µg/mg; Sigma-Aldrich, Steinheim, Germany) were used. All experiments were performed in triplicates in three independent experiments. For automated antimicrobial susceptibility testing of the clinical isolates, a Vitek®-2 system (Biomerieux Deutschland, Nürtingen, Germany) was used. Interpretation criteria for susceptibility and resistance were obtained from EUCAST. For the determination of resistance genes, PCR methods were employed [24,25].

### 4.3. Time-Kill Curves

Time-kill curves were performed according to the guideline of CLSI [26]. The detailed protocol has been described before [11]. Ranalexin, danalexin, cefuroxime (potency 88.6%) (Sigma-Aldrich, Steinheim, Germany) and physiological saline solution (growth control; Braun, Melsungen, Germany) were incubated with an adjusted inoculum (1 × 10^6^ cfu/mL) of *Staphylococcus aureus* ATCC 25923 or *Escherichia coli* ATCC 25922. After 0, 0.5, 1, 2, 4, 8 and 12 h, aliquots of 1× MIC, 2× MIC and 4× MIC were withdrawn, serially diluted in saline (1:10) and spread onto agar plates (Columbia agar with 5% sheep blood, Biomerieux Deutschland, Nürtingen, Germany). After 24 h of incubation at 37 °C, colonies on the plates were counted.

### 4.4. Radioactive Labeling and In Vivo Imaging

Male Wistar rats with a weight of 200–250 g were purchased from Janvier Labs (Saint-Berthevin Cedex, France) and kept at the animal facility of the Department of Nuclear Medicine until use for scintigraphic/PET imaging and biodistribution studies. For the animal experiments, approval was obtained from the Animal Welfare Board of the governmental office (Karlsruhe, Germany) and the University of Heidelberg Committee for Ethics on Laboratory Animal Experimentation, and testing was performed in compliance with the following institutional guidelines: the German law for animal protection, the Directive 2010/63/EU of the European Union on the protection of animals used for scientific purposes and FELASA (Federation of European Laboratory Animal Science Associations, Ipswich, UK) guidelines and recommendations.

The peptides were radiolabeled by the use of ^125^iodine (^125^I, Hartmann Analytic, Braunschweig, Germany) for scintigraphy studies. For the labeling procedure, the chloramine T method was used as described before [27]. Purification was achieved by preparative HPLC containing a Chromolith performance RP-18e that was equipped with a gamma detector [20]. ^68^Gallium (^68^Ga) was eluted from an iThemba LABS ^68^Ge/^68^Ga generator (DSD Pharma, Purkersdorf, Austria). Complexation of ^68^Ga with the chelator DOTA at pH 3.8 in acetate buffer and the subsequent purification of the labeled peptide were performed as previously described [20].

## 5. Conclusions

We were able to synthesize an all d-amino acid derivative of the AMP ranalexin with prolonged in vivo retention. Furthermore, the spectrum of antimicrobial activity and the antimicrobial kinetics remained constant upon exchange of the configuration of the amino acids. Therefore, the modification of AMPs with d-amino acids could be a step to overcome the disadvantages of natural AMPs such as proteolytic degradation and rapid excretion.

## Figures and Tables

**Table 1:** Amino acid sequences of the synthesized peptides.

Peptide	Amino Acid Sequence																			
Ranalexin	F	L	G	G	L	I	K	I	V	P	A	M	I	C	A	V	T	K	K	C
Ranalexin-d-Tyr	F	L	G	G	L	I	K	I	V	P	A	M	I	C	A	V	*y^1^*	K	K	C
Danalexin	*f*	*l*	G	G	*l*	*i*	*k*	*i*	*v*	*p*	*a*	*m*	*i*	*c*	*a*	*v*	*t*	*k*	*k*	*c*
d-Tyr-danalexin	*Y ^1^*	*l*	G	G	*l*	*i*	*k*	*i*	*v*	*p*	*a*	*m*	*i*	*c*	*a*	*v*	*t*	*k*	*k*	*c*
DOTA-d-Tyr-danalexin	*y*	*l*	G	G	*l*	*i*	*k*	*i*	*v*	*p*	*a*	*m*	*i*	*c*	*a*	*v*	*t*	*k*	*K ^2^*	*c*

The amino acid sequences are shown in one-letter code. d-amino acids are printed in lowercase. Basic amino acids are highlighted in blue. Cysteines (yellow) are linked via disulfide bonds. ^1^ Position of the coupling with ^125^iodine. ^2^ Position of the coupling with ^68^Ga-DOTA.

**Table 2:** Mass spectrometric analyses by HPLC-MS. Calculated mass, observed mass and interpretation of the identity of the detected species.

Peptide	Calculated Mass [Da]	Observed Mass [Da]	Detected Species
**Ranalexin**	2103.1890	2104.1768	[M + H]^+^
**Ranalexin-d-Tyr**	2165.0267	1083.6002	[M + 2H]^2+^
**Danalexin**	2103.1890	2104.1047	[M + H]^+^
**d-Tyr-danalexin**	2119.1839	2120.1700	[M + H]^+^
**DOTA-d-Tyr-danalexin**	2506.0098	2506.1562	[M]^+^

**Table 3:** Antibiograms of the clinical isolates.

	*A. baumannii* SC303336 4-MRGN	*A. baumannii* SC411190 4-MRGN, OXA-23	*E. faecium* UL407074 VanA	*K. pneumoniae* BL809453 4-MRGN, KPC
Amoxicillin/Clavulanic acid	nt	**R**	**R**	nt
Piperacillin	**R**	**R**	nt	**R**
Piperacillin/Tazobactam	**R**	**R**	nt	**R**
Cefuroxime	nt	nt	**R**	**R**
Imipenem	**R**	**R**	**R**	**R**
Meropenem	**R**	**R**	nt	**R**
Ciprofloxacin	**R**	**R**	**R**	**R**
Gentamicin	**R**	**R**	nt	**I**
Tobramycin	**R**	**R**	nt	**R**
Amikacin	**R**	**R**	nt	nt
Tigecycline	**I**	nt	**S**	**S**
Trimethoprim/Sulfamethoxazole	**R**	**R**	**R**	**R**
Vancomycin	nt	nt	**R**	nt
Teicoplanin	nt	nt	**R**	nt
Erythromycin	nt	nt	**R**	nt
Linezolid	nt	nt	**S**	nt
Colistin	**S**	**S**	nt	**S**

All isolates are multidrug-resistant (R-red). Only a few antibiotics remain active (S-green). *A. baumannii* SC303336, *A. baumannii* SC411190 and *K. pneumoniae* BL809543 belong to the 4-MRGN group. *E. faecium* UL407074 contains a vanA resistance gene. OXA-23 carbapenemases are produced by *A. baumannii* SC411190. *K. pneumoniae* produces a KPC-2 carbapenemase. I: intermediate (yellow), nt: not tested.

**Table 4:** Minimum inhibitory concentrations (MICs) of ranalexin, danalexin and positive controls against a representative selection of bacteria. Both peptides have strong antimicrobial activity against Gram-positive bacteria and Gram-negative bacteria of the genus *Acinetobacter*. Against other Gram-negative bacteria, only weak antimicrobial activity could be shown.

Bacterium	MIC [mg/L] (µM)		
	Ranalexin	Danalexin	Positive Control
**Gram-positive bacteria**			
*Bacillus megaterium* DSM 32	4 (1.9)	4 (1.9)	vancomycin 0.13
*B. subtilis* DSM 10	4 (1.9)	4 (1.9)	vancomycin 0.13
*Clostridium pasterianum* DSM 525	16 (7.6)	8 (3.8)	vancomycin 0.25
*Corynebacterium spheniscorum* DSM 44757	16 (7.6)	8 (3.8)	vancomycin 0.50
*Enterococcus casseliflavus* ATCC 700327 VanC ^1^	8 (3.8)	8 (3.8)	vancomycin 8
*E. faecalis* ATCC 29212	16 (7.6)	16 (7.6)	vancomycin 1
*E. faecium* UL407074^2^ VanA ^3^	16 (7.6)	8 (3.8)	vancomycin 640
*Staphylococcus aureus* ATCC 25923	8 (3.8)	4 (1.9)	vancomycin 1
*S. aureus* NCTC 10442 MRSA ^4^	8 (3.8)	8 (3.8)	vancomycin 1
*S. epidermidis* ATCC 14990	16 (7.6)	16 (7.6)	vancomycin 2
*S. saprophyticus* ATCC 15305	8 (3.8)	16 (7.6)	vancomycin 2
**Gram-negative bacteria**			
*Acinetobacter baumannii* SC303336^2^ 4-MRGN ^5^	4 (1.9)	4 (1.9)	colistin 0.25 ^6^
*A. baumannii* SC322333^2^ 4-MRGN ^5^	8 (3.8)	16 (7.6)	colistin 1 ^6^
*A. baumannii* SC411190^2^ 4-MRGN ^5^	4 (1.9)	8 (3.8)	colistin 0.25 ^6^
*Escherichia coli* ATCC 25922	32 (15.2)	32 (15.2)	colistin 0.25 ^6^
*E. coli* 0157:H7 ATCC 35150 EHEC ^7^	32 (15.2)	32 (15.2)	colistin 0.50 ^6^
*Klebsiella pneumoniae* ATCC 700603	>64 (>30.4)	>64 (>30.4)	colistin 1 ^6^
*K. pneumoniae* BL809453 ^2^	>64 (>30.4)	>64 (>30.4)	colistin 0.25 ^6^
*Pseudomonas aeruginosa* ATCC 27853	64 (30.4)	64 (30.4)	colistin 0.25 ^6^
*P. fluorescens* DSM 50090	>64 (>30.4)	>64 (>30.4)	doxycycline 0.50
*Yersinia mollaretii* DSM 18520	>64 (>30.4)	>64 (>30.4)	colistin 0.25 ^6^

^1^*Enterococcus casseliflavus* ATCC 700327 possesses a natural low-level resistance against vancomycin, ^2^ Clinical isolate of Heidelberg University Hospital, ^3^ *E. faecium* UL407074 possesses a high-level resistance against vancomycin of the type vanA, ^4^ Methicillin-resistant *Staphylococcus aureus*, ^5^ Multidrug-resistant Gram-negative bacteria against four different groups of antibiotics, ^6^ The MICs of colistin are lower than expected, due to the use of non-absorbent material [12], ^7^ Enterohemorrhagic *Escherichia coli*.
